# Stabilizing the Convergence of Pixel-Based Deep Active Inference Controllers Using Adaptive Smoothing Filters

**DOI:** 10.3390/biomimetics11010001

**Published:** 2025-12-19

**Authors:** Kazuma Nagatsuka, Kyo Kutsuzawa, Dai Owaki, Mitsuhiro Hayashibe

**Affiliations:** Department of Robotics, Graduate School of Engineering, Tohoku University, Sendai 9808579, Miyagi, Japan; nagatsuka.kazuma.s8@alumni.tohoku.ac.jp (K.N.); kutsuzawa@mail.saitama-u.ac.jp (K.K.); owaki@tohoku.ac.jp (D.O.)

**Keywords:** active inference, free energy principle, robotics, machine learning

## Abstract

In recent years, active inference has gained attention in robot control owing to its adaptability to environmental changes. However, its reliance on gradient descent of variational free energy offers no guarantee of convergence to an optimal solution. In this study, we propose an approach that applies a smoothing filter to a pixel-based active inference controller to mitigate the risk of local minima. By smoothing the observed, predicted, and target values, the free energy function becomes smoother, yielding a broader distribution of gradients toward the target, thereby reducing the risk of being trapped in the local minima. In addition, in order to prevent excessive smoothing from eliminating the gradient of the free energy function, we also proposed a method for dynamically adjusting the intensity of smoothing based on prediction and target errors. To evaluate the effectiveness of our method, we applied it to two simulation environments: a simple object-tracking task using a 3-degrees-of-freedom camera, and a robot control task using a 2-degrees-of-freedom robotic arm, and compared it with the conventional active inference controller as a baseline. The experimental results demonstrate that the proposed approach achieves improved convergence performance over the conventional method.

## 1. Introduction

Most conventional robot control methods rely on accurate environmental models. However, in dynamically changing environments, model inaccuracies can lead to significant performance degradation. Therefore, numerous learning-based approaches, such as imitation learning [1] and reinforcement learning [2] have been proposed. However, these methods still face challenges, including the need for iterative trial and error and the collection of multiple data [3]. By contrast, active inference has recently emerged as a promising framework for online adaptation, offering an alternative that mitigates the limitations of traditional learning-based methods.

Active inference is a mathematical framework for modeling the brain that is recognized in the field of neuroscience [4]. In this framework, it is assumed that humans possess an internal model that represents the generation of sensory data [5]. In active inference, both state estimation and biological system behavior are explained through the minimization of a single objective function, known as the variational free energy, which quantifies the discrepancy between predictions generated by an internal model and observed sensory data. Specifically, the idea of active inference is to consider the control of bodily movements in animals as the fulfillment of prior expectations of proprioceptive sensations. It has increasingly been recognized that active inference provides a comprehensive explanation of various neural mechanisms [5,6].

Recently, active inference has gained attention in the field of robotics owing to its adaptability to environmental changes and robustness to noise [7]. In robotics, internal state estimation is a critical topic because it is necessary to infer the correct internal state from sensory data [8]. Active inference updates state estimates in real time using a gradient descent on free energy, providing robustness to noise and sensory fluctuations. Meanwhile, reinforcement learning and other machine learning-based methods have gained attention for generating robotic movements [3]. Among these methods, research on their robustness to environmental variations has been actively pursued [9,10]. However, these machine-learning-based approaches, particularly in real-world environments, often incur significant costs in collecting training data. The Active Inference Controller (AIC) proposed by Pezzato et al. [11] applies active inference to robot control, where both the estimated states and control inputs are updated via a gradient descent on the variational free energy as the objective function. The AIC has demonstrated strong robustness to observational noise and unexpected variations in sensory data. Other studies have explored the application of active inference in robot control [12,13,14,15]. Furthermore, Sancaktar et al. [16] proposed the pixel-based deep Active Inference (PB-AIC), which is an extension of the AIC designed for scenarios in which only visual input is available. Meo et al. [17] introduced a Multimodal Active Inference Controller (M-AIC), that leverages a multimodal variational autoencoder to incorporate multiple sensory modalities as observations.

However, because the AIC relies on gradient descent with variational free energy as an objective function, convergence to the optimal solution is not guaranteed, and may result in entrapment in local minima [6]. In particular, the PB-AIC has been reported to fall into local minima unless the initial posture is close to the target posture [16]. Furthermore, whereas M-AIC improves adaptability and robustness by utilizing multimodal observations, it has been found that excluding visual information results in smoother movement control [17]. Conventional AICs use low-dimensional sensory information, including joint angles as observations, resulting in fewer occurrences of local minima in the free energy function. Consequently, the issue of local minima in continuous-time active inference controllers has not been extensively discussed. Most studies have focused on action planning in discrete-time settings [6,18]. However, there are scenarios where joint angle information may become unavailable owing to sensor failures or where M-AIC may require the integration of various observational modalities beyond joint angles. In these cases, the use of visual information is crucial. Moreover, vision sensors are inherently advantageous for robotic applications because they allow non-contact environmental measurements [19].

Therefore, Active Inference using visual observations’ tendency to become trapped in local minima remains a significant issue that cannot be ignored. To address this issue, this study makes the following contributions. First, we propose a method for avoiding local minima by smoothing image observations to reduce fine features and smooth edges, thereby expanding the gradient basin toward the visual target. Second, because excessive smoothing can remove important features and cause divergence, we introduce an adaptive mechanism that adjusts the smoothing strength over time to balance robustness to local minima and convergence accuracy. Third, we apply the proposed method to two environments—a 3-DOF camera-based object-tracking task and a 2-DOF robot-arm control task—and empirically evaluate its performance against conventional AIC/PB-AIC baselines.

## 2. Active Inference Framework

### 2.1. The Free Energy Principle

The Free Energy Principle, proposed by Friston et al. [4] in the field of neuroscience, provides a theoretical framework that reformulates Helmholtz’s idea [20] that perception involves inferring the structure of the external world from sensory signals—within a Bayesian inference framework. This explains perception as equivalent to the minimization of a single objective function known as free energy. The Free Energy Principle can be applied to state estimation in robotics. Fundamentally, a robot does not have direct access to its true state, and must infer it from noisy observations. For example, when measuring joint angles using a rotary encoder, noise is inevitably introduced in real-world environments, necessitating the estimation of the true joint angle. Such state estimation can be formulated as an optimization problem, where the free energy serves as a single objective function.

As a robot does not have direct access to its true state, we define *z* as the state to be estimated and *o* as the observed data. The goal is to determine the posterior distribution p(z|o), which represents the probability of the state, provided the observation. According to Bayes’ theorem, this can be expressed as

(1) $$\begin{array}{c} p\left(z|o\right)=\frac{p(o|z)p(z)}{p(o)} \end{array}$$

where p(o|z)p(z)=p(o,z) represents the internal model assumed to be known. However, computing p(o) requires integration over all the possible states

(2) $$\begin{array}{c} p(o)=\int p(o|z)p(z)\;dz \end{array}$$

which, in most cases, are computationally intractable. Therefore, variational Bayesian inference is used to approximate the true posterior p(z|o) by minimizing the Kullback (KL) divergence between the true posterior and the approximate distribution [21]. The KL divergence quantifies the difference between two probability distributions, reaching 0 when the distributions are identical and positive otherwise. Thus, minimizing the KL divergence ensures that the approximate distribution closely resembles the true posterior distribution. Let q(z) denote the approximate distribution, the KL divergence for continuous probability distributions is expressed as:

(3) $$\begin{array}{c} KL\left[q\left(z\right)||p\left(z|o\right)\right]=\int_{z}q\left(z\right)\ln\frac{q(z)}{p(z|o)} \end{array}$$

The variational free energy is defined as follows:

(4) $$\begin{array}{c} F=\int_{z}q\left(z\right)\ln\frac{q(z)}{p(z|o)}-\ln p\left(o\right) \end{array}$$

The term −lnp(o) is known as surprise [21]. The surprise term depends only on the sensory signal *o*. When the sensory signal is static, minimizing the KL divergence is equivalent to minimizing the free energy. Therefore, to reduce the KL divergence, it is necessary to minimize the free energy. The system can accurately infer the true state by minimizing the free energy.

Although the surprise term depends only on sensory signals, these signals can be influenced by an agent’s actions, which modify its relationship with the environment. Therefore, the sensory signal *o* can be considered a function of the agent’s action, *a*. Therefore, when an agent’s predictions deviate from its observations, it can refine its state estimates to improve predictions and actively interact with the environment to adjust observations. Because the KL divergence is non-negative, the surprise term is bounded above by the free energy. Thus, minimizing free energy also minimizes surprise.

Therefore, the agent actively interacts with the environment to modify the sensory signals and reduce free energy, a process known as active inference. Consequently, both state estimation and action selection can be formulated using gradient descent, with free energy as the objective function.

(5) $$\begin{array}{c} z=\underset{z}{\text{argmin}}F\left(o,z\right) \end{array}$$

(6) $$\begin{array}{c} a=\underset{a}{\text{argmin}}F\left(o(a),z\right) \end{array}$$

### 2.2. Active Inference Controller

The AIC applies the principles of active inference described above to robot control. In robot control, internal states, such as joint angles, are observed using sensors such as rotary encoders. The system must estimate the true state while simultaneously controlling the robot to achieve the target value. Thus, the internal model consisting of p(o|z) and p(z) can be formulated as follows under the assumption that the noise terms *r* and *w* follow Gaussian distributions:

(7) $$\begin{array}{c} o=g(z)+r \end{array}$$

(8) $$\begin{array}{c} Dz=f(z)+w \end{array}$$

where Dz is the time derivative of *z*. In the AIC proposed by Pezzato et al. [11], the joint angles were used as observations. Therefore, the system is formulated such that the observation model follows an identity function with added noise, as expressed by the following equation:

(9) $$\begin{array}{c} \mu =g(\mu )+r \end{array}$$

(10) $$\begin{array}{c} D\mu =\mu_{d}-\mu +w \end{array}$$

where μ represents the joint angle beilef and $\mu_{d}$ is the target joint angle. The dynamics of μ, denoted by Dμ, are formulated under the assumption that the internal model has prior knowledge that μ should converge toward the target joint angle $\mu_{d}$. Based on this assumption, the AIC is formulated as follows:

(11) $$\begin{array}{c} \frac{d\mu }{dt}=D\mu -\frac{\partial F}{\partial \mu } \end{array}$$

(12) $$\begin{array}{c} \frac{da}{dt}=-\frac{\partial F}{\partial a} \end{array}$$

### 2.3. Pixel-Based Deep Active Inference Controller

The Pixel-Based Deep Active Inference Controller (PB-AIC) is an extension of the previously described AIC to accommodate image-based observations. While the estimated internal states, such as the joint angles, remain the same, the key difference is that the observations consist of images rather than direct joint angle measurements. In PB-AIC, the internal model is formulated as follows:

(13) $$\begin{array}{c} x_{v}=g\left(\mu \right)+r \end{array}$$

(14) $$\begin{array}{c} D\mu =T(\mu )\beta (\rho -g(\mu ))+w \end{array}$$

where $T\left(\mu \right)=\partial g{\left(\mu \right)}^{T}/\partial \mu$ denotes mapping from the image to the joint angle, $x_{v}$ is the predicted image, ρ denotes the target image, and β denotes the gain parameter. Because predicting images corresponding to internal states is necessary, Sancaktar et al. [16] utilized the decoder of a Variational Autoencoder (VAE). Similarly, in this study, we employed a decoder model to generate images from internal states.

In this study, we applied the PB-AIC to two environments: a 3-DOF camera-based object tracking environment and a 2-DOF robot arm control environment. For the robot arm environment, we followed the implementation of Sancaktar et al. [16] and Oliver et al. [12], using the joint angular velocity as the control input and applying the same approximation. Thus, the update equations for the state and control inputs can be expressed as follows:

(15) $$\begin{array}{c} \dot{\mu }=k_{v}\frac{\partial g{\left(\mu \right)}^{T}}{\partial \mu }\Sigma_{v}^{-1}\left(x_{v}-g\left(\mu \right)\right)+\frac{\partial f{\left(\mu ,\rho \right)}^{T}}{\partial \mu }\Sigma_{\mu }^{-1}\left(-f\left(\mu ,\rho \right)\right) \end{array}$$

(16) $$\dot{a}=-\frac{\partial g{\left(\mu \right)}^{T}}{\partial \mu }\Delta_{t}\Sigma_{v}^{-1}\left(x_{v}-g\left(\mu \right)\right)$$

where $\Sigma_{v}$ and $\Sigma_{\mu }$ are the variances of Gaussian distributions *r* and *w*, respectively, and $k_{v}$ is a parameter that adjusts the update balance between state estimation and control. Assuming that the time interval of the control cycle is Δt and that the true states $q_{j}$ and belief $\mu_{j}$, as well as $x_{v}$ and g(μ), converge to equilibrium, the following approximations are employed:

(17) $$\begin{array}{c} \frac{\partial q_{i}}{\partial a_{i}}=\frac{\partial \mu_{i}}{\partial a_{i}}=\Delta t \end{array}$$

(18) $$\begin{array}{c} \frac{\partial x_{v}}{\partial a_{i}}=\frac{\partial x_{v}}{\partial q_{i}}\frac{\partial q_{i}}{\partial a_{i}}=\frac{\partial g(\mu )}{\partial \mu_{i}}\frac{\partial \mu_{i}}{\partial a_{i}} \end{array}$$

In the camera environment, posture changes are directly used as control inputs to enable stable position control. This equation is formulated as follows:

(19) $$\frac{\partial q_{i}}{\partial a_{i}}=\frac{\partial \mu_{i}}{\partial a_{i}}=1$$

## 3. Proposed Method

**Figure 1:**
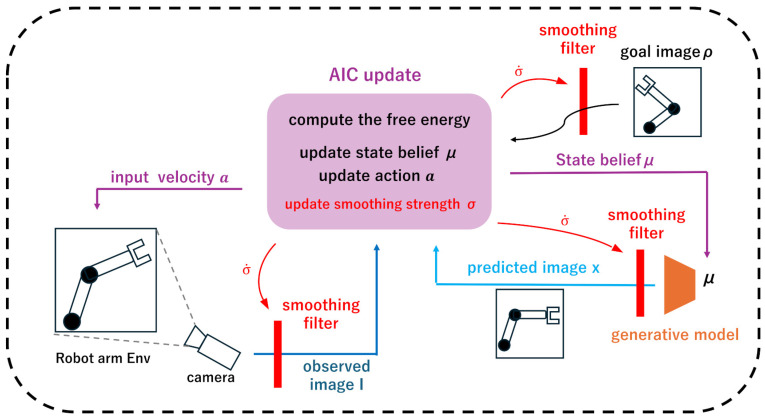
Overview architecture of our proposed method.

An overview the architecture of the proposed method is shown in Figure 1. Our method follows the architecture of the Pixel-Based Deep Active Inference Controller (PB-AIC). As our approach primarily involves the addition of an image-smoothing layer, its implementation is straightforward. In this study, we applied the proposed method to two environments: (1) a 3-DOF camera environment, where a camera is manipulated to center an object in its field of view and (2) a 2-DOF robot-arm environment in which a robotic arm is controlled.

### 3.1. Image Smoothing

Thus far, we have explained the theory and implementation of the existing PB-AIC. However, as aforementioned, it tends to become trapped in the local minima unless the initial posture is close to the target. Therefore, we propose introducing an image-smoothing layer. By softening image edges, this layer helps mitigate the issue of local minima. This section provides a detailed description of the proposed image-smoothing layer.

Image smoothing is a fundamental technique in image processing that is used to remove noise and blur images, while preserving structural information and smoothing edges. By applying image smoothing, various image-processing tasks, such as saliency estimation [22], edge detection [23], and non-photorealistic rendering [24] can be performed more efficiently.

Image-smoothing methods are categorized into filtering-based and optimization-based approaches [25]. Filtering-based methods [26,27,28] apply a filter to an input image and compute the output pixel values as a weighted average of the input pixels within the filter. By contrast, optimization-based methods [29,30] formulate image smoothing as an optimization problem, often outperforming filtering-based approaches in suppressing artifacts. However, a drawback of optimization-based smoothing is its high computational cost, owing to the need for global optimization.

Various smoothing filters exist; however, but in this study, we employed a simple Gaussian filter. The Gaussian filter is widely used for image smoothing [23] and its a filter whose weights follow a Gaussian distribution. By performing a convolution operation between the filter and image, the image can be smoothed.

The smoothing intensity of the Gaussian filter can be adjusted by modifying either the filter size or standard deviation of the Gaussian distribution. The Gaussian filter is mathematically expressed as follows: x,y represents the pixel positions in the image, and σ denotes the standard deviation of the Gaussian distribution [23]:

(20) $$G\left(x,y\right)=\frac{1}{2\pi \sigma^{2}}\exp\left(-\frac{x^{2}+y^{2}}{2\sigma^{2}}\right)$$

In this implementation, a smoothing filter is applied to the observed, predicted, and target images.

### 3.2. Updating Smoothing Strength

The smoothing intensity of the image can be adjusted by changing the standard deviation of the Gaussian filter. As discussed later, excessive smoothing reduces the image features, causing the gradients to vanish. Therefore, we propose a method that dynamically adjusts the smoothing intensity based on image errors. Specifically:

- For perception, the smoothing intensity was updated using the error between the observed and predicted images.
- For active inference, both the prediction and target errors were used for updating.

Mohamed et al. [31] previously updated the parameters using free energy; however, because computing the partial derivative of free energy with respect to σ is challenging, we approximate the update equation as follows:

(21) $$\dot{\sigma }=-\frac{1}{m({\left(x_{v}-g\left(\mu \right)\right)}^{2}+{\left(\rho -g\left(\mu \right)\right)}^{2})}$$

where *m* is the update rate time constant. For perception,

(22) $$\dot{\sigma }=-\frac{1}{m({\left(x_{v}-g\left(\mu \right)\right)}^{2})}$$

When prediction deviates significantly from observation and target values, strong smoothing is necessary to prevent the system from becoming trapped in the local minima. Ideally, the smoothing intensity gradually decreases as the error diminishes. Consequently, the update equation for smoothing intensity can be approximated as the reciprocal of a term similar to the free energy, as expressed in Equations (21) and (22). The update rule for the smoothing intensity in Equations (21) and (22) is introduced as an engineering heuristic that approximates the inverse of the estimated free energy, since the exact partial derivative is difficult to compute in our setting. Therefore, this rule is not fully consistent with the original formulation of the Free Energy Principle.

## 4. Experiments

In this study, the proposed method was applied to two environments: camera and robot arm environments. The key distinction lies in visibility—the controlled object (robot arm) is visible in the image in the robot arm environment, whereas in the camera environment, the controlled object (camera) is not directly visible. The camera environment was a simplified setting that returned images for a particular posture, while the robot arm environment employed a more realistic simulator to emulate real-world conditions. First, in the camera environment, we conducted statistical comparisons between cases with and without smoothing. Furthermore, we evaluated the performance when the smoothing intensity was dynamically adjusted based on image error. Subsequently, in the robot-arm environment, we conducted statistical comparisons between cases with and without image smoothing. In addition, we evaluated the performance when the smoothing intensity was adjusted dynamically, as in the camera environment.

### 4.1. Generative Model

The structure of the decoder model used in the experiment is listed in Table 1. The model comprises three types of layers: fully connected (FC), convolutional (Conv), and transposed convolutional (upConv). The initial input dimension is 2 in the robot arm environment (corresponding to the number of joints) and 3 in the camera environment (corresponding to the camera posture).

In the camera environment, the decoder model was trained on a dataset of images containing the target object along with the corresponding camera poses. A total of 70,000 data samples were used for training. The data collection environment was identical to that used in the experiment. The dataset was generated by collecting images corresponding to camera poses sampled from a uniform distribution within a specified range (30≤x≤98,30≤y≤98,0≤θ<2π). However, in the robot arm environment, the decoder model was trained using joint angles and their corresponding images as the training dataset. A total of 10,000 data samples were employed for training. The environment used for data collection was the same as that employed in the robot arm experiments but with an AIC operating at a time step of 0.0001 s. The simulation environment was designed such that every 1000 loops, a new random target value was set. During the simulation, the images and their corresponding joint angles were recorded every 100 loops to construct a dataset. The target values were restricted to the range of −2.2 rad to 2.2 rad.

### 4.2. Camera Environment

#### 4.2.1. Experimental Setup in the Camera Envrionment

In this experiment, we prepared a simplified simulation environment, in which an input camera pose (x,y,θ) returned the corresponding image. The task involved controlling the camera pose such that the captured image matched the target image, ensuring that the object was centered in the camera’s field of view. The object was set as a simple isosceles triangle. Although a frame is visible in the image, it is part of the camera and remains fixed in position and orientation, even when the camera moves. The simulation time step was set to 0.001 s, and the simulation was terminated when both conditions were met for 1 s (i.e., 1000 steps):

- The L2 norm between the current and target values was lower than 0.15.
- The orientation error θ was lower than 0.01.

These threshold values and number of steps were determined empirically based on the observed values, indicating convergence.

**Figure 2:**
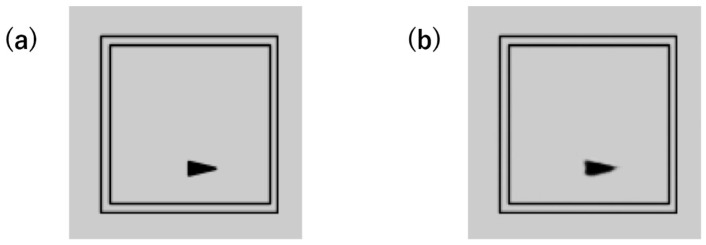
(**a**) Camera observation image example. (**b**) Camera prediction image example.

If these conditions were not satisfied, the simulation was continued for a maximum of 25 s (i.e., 25,000 steps). The training data consisted of camera movements within the range of 30≤x≤98,30≤y≤98,0≤θ<2π[rad]; therefore, the camera movement was restricted to this range. The observation received was 128×128 grayscale image, in which the object was visible. Figure 2 displays an example of an observed image and an example of image prediction from the trained model.

In this experiment, the size of the smoothing filter was fixed at 41×41. Each image was smoothed and then normalized in the range of 0–1. The kernel size of the Gaussian filter was fixed to 41×41 in all experiments. This size was chosen empirically as a trade-off between capturing sufficient spatial context and computational cost. For the typical standard deviation 7 used in our experiments, a 41×41 kernel roughly covers the effective support of the Gaussian (≈3σ) while keeping the convolution feasible on 128×128 images. In addition, the simulation was executed in different environments depending on the presence of smoothing. When no smoothing was applied, the simulation was performed on a CPU environment. By contrast, when smoothing was applied, the simulation was executed in a GPU environment to accelerate the convolutional computations.

#### 4.2.2. Camera Environment Results

In this experiment, the target image was fixed at postures x=64, y=64, and θ=π/2. The initial joint angles were randomly generated for each of the following four levels, with 25 samples per level, resulting in 100 trials (25 trials per level).

- Level 1: Random positions on a circle of radius three around the target position, with angles offset from the target angle by either +π/24 or −π/24.
- Level 2: Random positions on a circle of radius 12 around the target position, with angles offset from the target angle by either +π/15 or −π/15.
- Level 3: Random positions on a circle of radius 18 around the target position, with angles offset from the target angle by either +π/12 or −π/12.
- Level 4: Random positions on a circle of radius 24 around the target position, with angles offset from the target angle by either +π/6 or −π/6.

As the distance from the target position increases (i.e., as the level increases), the convergence becomes more challenging. In this experiment, the Gaussian filter was set with a filter size of 41 and a standard deviation of 7. The parameters used for the active inference controller in this setting are listed in Table 2. To enhance the convergence performance further, separate parameter values were employed for the *x*-position, *y*-position, and angle θ.

**Figure 3:**
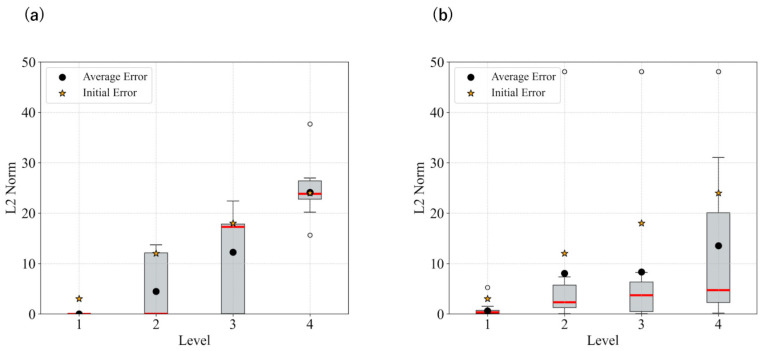
Statistical comparison of L2 norm in the camera environment: (**a**) No filters applied; (**b**) Filter applied: sigma = 21.

**Figure 4:**
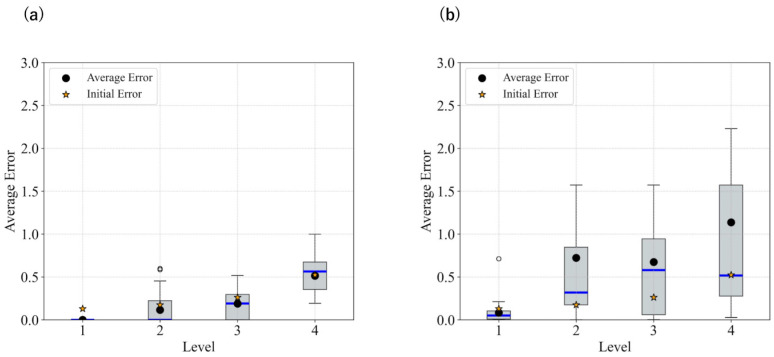
Statistical comparison of theta error in the camera environment: (**a**) No filters applied; (**b**) Filter applied: sigma = 21.

The statistical results for each level in the case without smoothing, as well as those with smoothing, are shown in Figure 3 and Figure 4. Figure 3 presents the L2 norm results only calculated based on x,y coordinates, while Figure 4 shows the absolute error results for θ.

These results show that image smoothing helped maintain the L2 norm relatively low; however, instances of divergence—characterized by a sudden increase in the L2 norm without recovery—were still observed. Additionally, for θ, the performance deteriorated when smoothing was applied.

**Figure 5:**
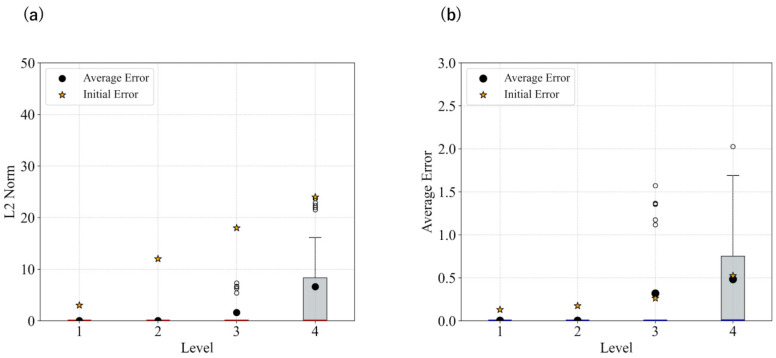
Statistical results when updating the smoothing intensity in the camera environment: (**a**) L2 norm result; (**b**) Theta error result.

Figure 5 presents the statistical results for each angular deviation from the target when using updates with m=0.025. The smoothing strength was updated from 7 to 1. The time constant parameter, *m* and smoothing intensity were manually adjusted. The results indicate that both the L2 norm and θ error are effectively reduced, and divergence is suppressed.

**Figure 6:**
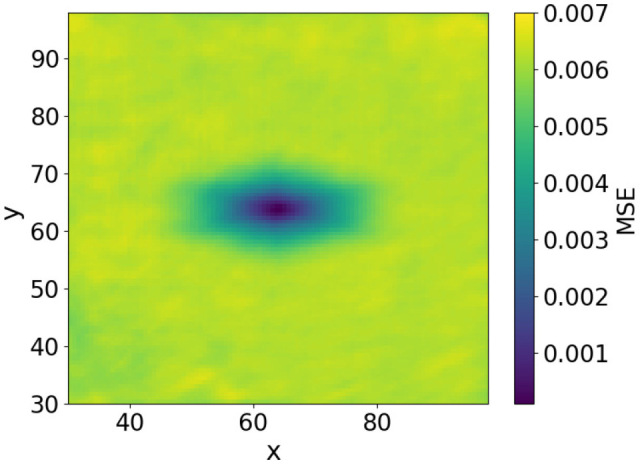
Heatmap plot of MSE in the camera environment (No filters applied).

**Figure 7:**
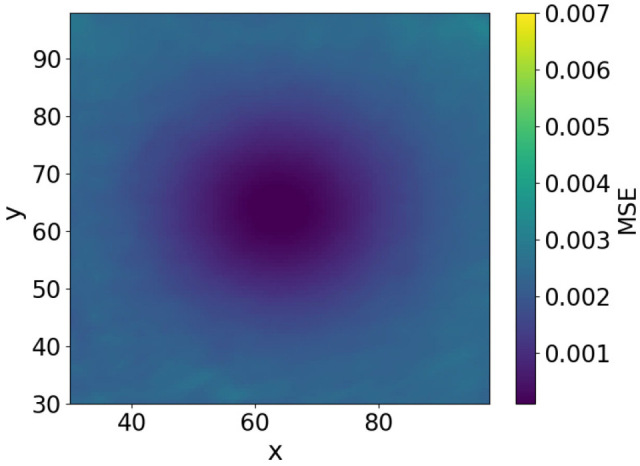
Heatmap plot of MSE in the camera environment (Filter applied: sigma = 7).

**Figure 8:**
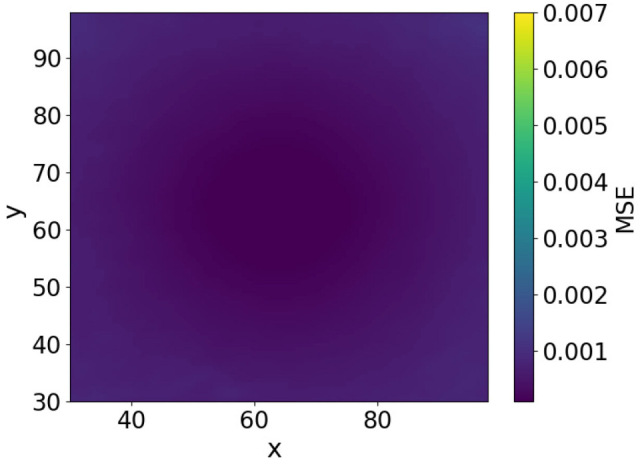
Heatmap plot of MSE in the camera environment (Filter applied: sigma = 19).

To further investigate the effect of image smoothing, we analyzed the method by plotting the MSE heatmaps for three cases: without smoothing, with smoothing at σ=7, and with smoothing at σ=19. The MSE of image approximately represents the free-energy function. The heatmaps in Figure 6, Figure 7 and Figure 8 show the MSE between the predicted and target images while varying *x* and *y* within the control range. From these results, we observe that as the smoothing intensity increases, the region where the gradients emerge expands.

**Figure 9:**
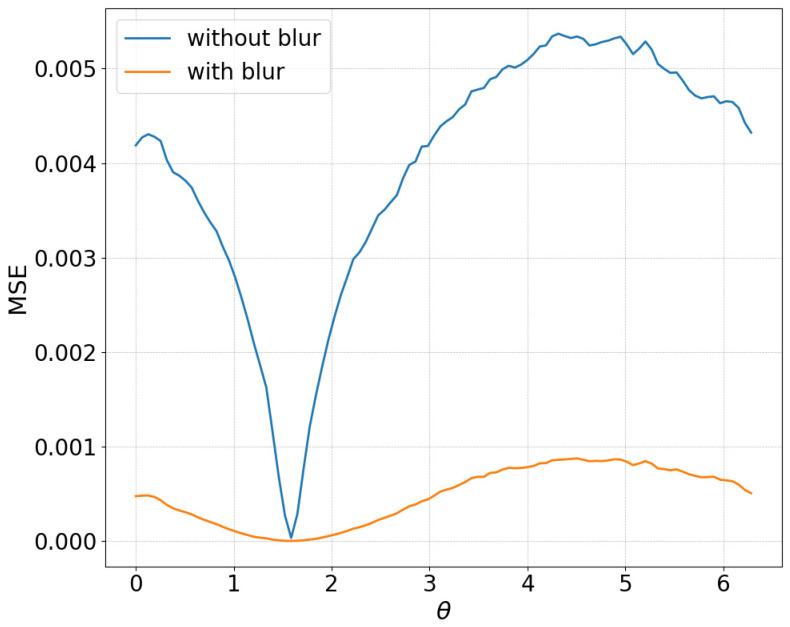
MSE plot for theta in the camera environment without blur and with blur (sigma = 7).

However, as shown in Figure 9, when plotting the MSE with respect to only θ in a situation where the object is located at the center of the image, the number of local minima in θ was originally small, resulting in minimal change.

### 4.3. Robot Arm Environment

#### 4.3.1. Experimental Setup in the Robot Arm Environment

For the robot arm environment, we used MuJoCo [32] as the simulator, with a 2-DOF robot arm as the controlled object. The task was formulated as a reaching task, in which the goal was to move the robotic arm to the target posture. The simulation time step was set to 0.005 s, and the simulation was terminated if the L2 norm between the current and the target values remained below 0.1 for 1 s (i.e., 200 steps). These threshold values and number of steps were determined empirically based on the observed values, indicating convergence. If this condition was not satisfied, the simulation was allowed to continue for a maximum of 150 s (i.e., 30,000 steps). The training data consisted of movements within the range of −2.2 rad to 2.2 rad; thus, the robot arm’s joint motion was restricted to this range.

**Figure 10:**
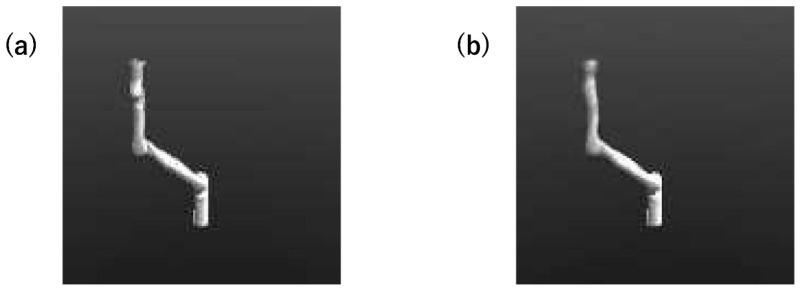
(**a**) Arm observation image example. (**b**) Arm prediction image example.

The observation received was a 128×128 grayscale image that captured the entire robot arm. To facilitate the training of the predictive model, the background of the images was set simple with a uniform black color. Examples of the observed image and the image generated from the trained model are shown in Figure 10.

In this study, the smoothing filter size was fixed at 41×41, and each image was normalized to a range of 0 to 1 after smoothing. Simulations were executed in different computing environments depending on the presence of smoothing: CPU for simulations without smoothing and GPU for those with smoothing to accelerate the convolutional computations.

#### 4.3.2. Robot Arm Environment Results

First, we conducted a statistical comparison of the cases in which only a smoothing layer was added. Subsequently, we statistically analyzed the performance when the smoothing intensity was dynamically updated.

For this analysis, we randomly generated target values following a Gaussian distribution and statistically analyzed the L2 norm at the end of the simulation. We considered initial angular deviations from the target values of 0.6 rad, 0.8 rad, 1.0 rad, 1.2 rad, 1.4 rad, and 1.6 rad, and performed comparisons for each case. As the initial angular deviation from the target increases, the difficulty of converging to the target increases. For each initial deviation, 12 poses were generated, resulting in 72 trials. In this experiment, a Gaussian filter with a filter size of 41 and a standard deviation of 21 was used. The parameters used for the active inference controller in this setting are listed in Table 3. To enhance the convergence performance further, different parameter values were employed for each joint.

**Figure 11:**
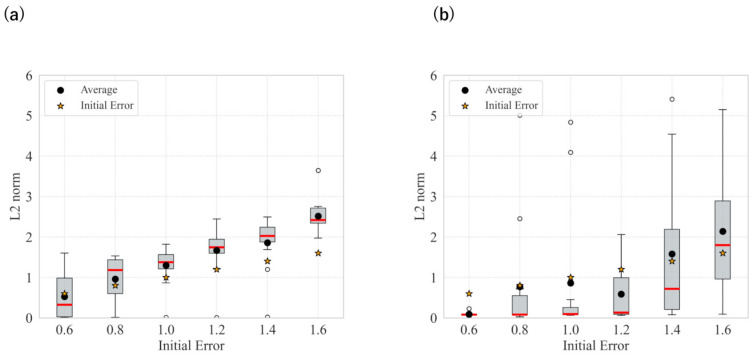
Statistical comparison of L2 norm in the robot arm environment: (**a**) No filters applied; (**b**) Filter applied: sigma = 21.

The statistical results for each angular deviation from the target without smoothing, as well as the results with smoothing, are shown in Figure 11. Similar to the results for the camera environment, we found that image smoothing generally helped maintain a relatively low L2 norm. However, there were various cases in which the system deviated further from the target compared to the unsmoothed case.

**Figure 12:**
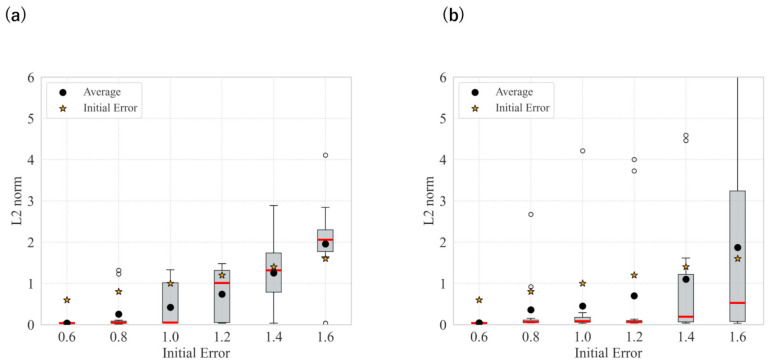
Statistical result when updating the smoothing intensity in the robot arm environment: (**a**) *m* = 0.005; (**b**) *m* = 0.05.

The statistical results for each angular deviation from the target with updates at m=0.005 and m=0.05 are shown in Figure 12. The smoothing strength was updated from 21 to 3. The time constant parameter, *m* and smoothing intensity were manually adjusted. Observing the results for m=0.005, we found that the L2 norm was relatively low while effectively suppressing divergence. In addition, as shown in Figure 12, when the update speed was set to a lower value, the number of diverging cases increased. However, the number of successfully converging cases also increased.

**Figure 13:**
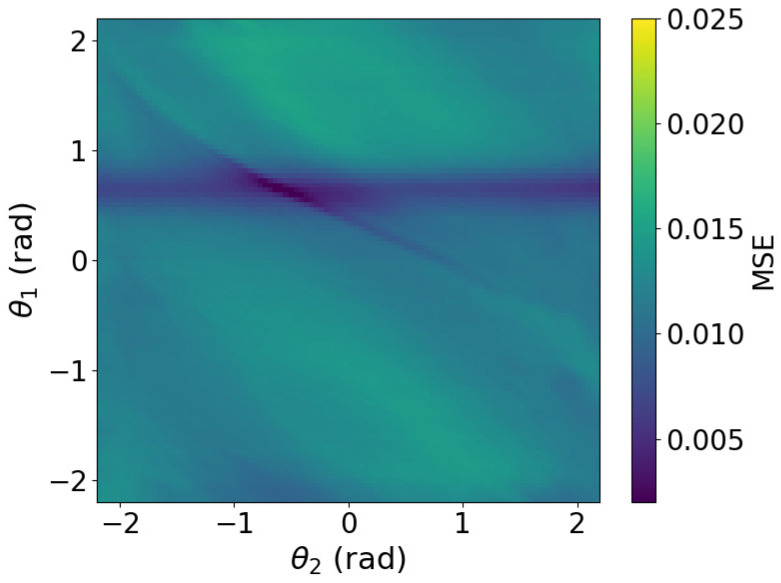
Heatmap plot of MSE in the robot arm environment (No filters applied).

**Figure 14:**
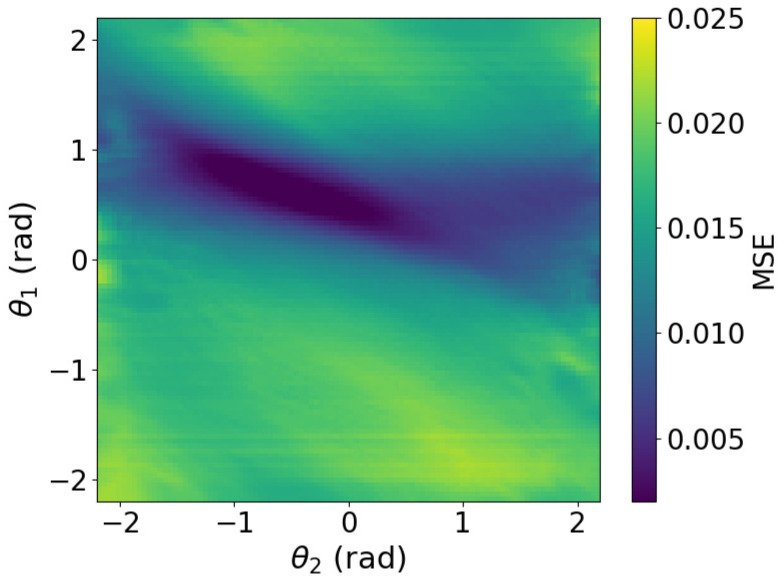
Heatmap plot of MSE in the robot arm environment (Filter applied: sigma = 7).

**Figure 15:**
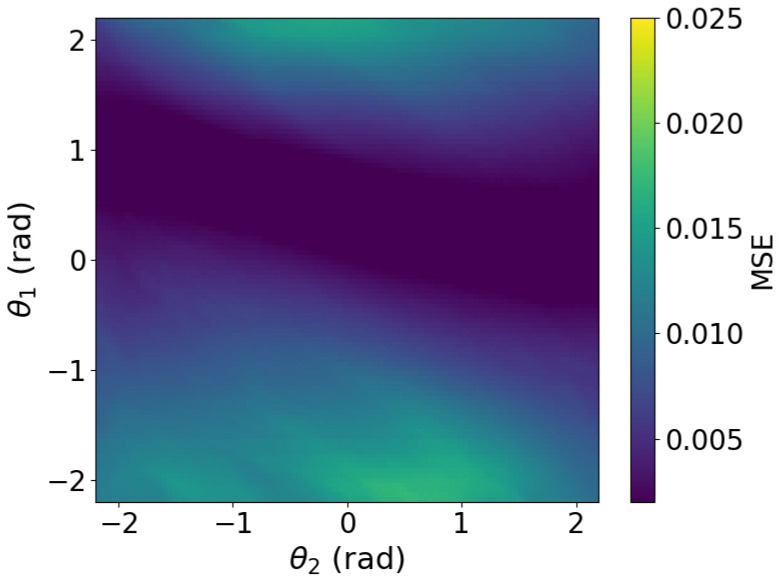
Heatmap plot of MSE in the robot arm environment (Filter applied: sigma = 19).

We analyzed MSE between the predicted and target images under three conditions: without smoothing, with smoothing at σ=7, and with smoothing at σ=19. The image MSE approximates the free-energy function. Heatmaps in Figure 13, Figure 14 and Figure 15 plot MSE errors across first and second joint angles from −2.2 rad to 2.2 rad. The results show that increasing the smoothing intensity expands the region in which gradients emerge.

## 5. Discussion

In both the camera and robot arm environments, the proposed method successfully avoided local minima in most cases. In contrast to the existing method, which struggle to converge as the gradient diminishes when the state is far from the target value, the proposed method achieved convergence in most cases as shown in Figure 3, Figure 4 and Figure 5. For example, even for the most difficult initial condition (level 4), the median final L2 norm decreased from approximately 25 to almost 0 with the proposed method in the camera environment. In the robot-arm environment, for initial deviations up to 1.4, the median final L2 norm similarly decreased from about 2 to nearly 0. As illustrated in Figure 8 and Figure 15, the gradient’s effective range is expanded, indicating that the proposed method effectively addresses the limitations of conventional approach.

Despite these advantages, the maximum image error varies depending on the smoothing intensity, which may affect convergence performance. In particular, increasing the smoothing intensity reduced the overall image error. This effect occurs because stronger smoothing removes finer image features [25], leading to the loss of detailed information. Consequently, because the Pixel-Based Active Inference directly connects the overall image error to the gradient descent update step size, smoothing the image tends to slow the convergence speed. Figure 3 and Figure 11 show that applying smoothing can lead to divergence in some cases. While increasing the smoothing intensity helps prevent local minima, excessive smoothing expands the region where the gradient vanishes, as shown in Figure 8, hindering convergence of the estimated states. In addition, in the camera environment, the control accuracy of the rotational angle θ decreased, as shown in Figure 4. This decline was probably owing to the loss of fine features from smoothing. For example, even in humans, excessive blurring makes it difficult to determine the orientation. Similarly, in the robot-arm environment, increasing the smoothing intensity reduced the gradient of the second joint as shown in Figure 15. Collectively, these results demonstrate a trade-off between smoothing strength and effective state estimation.

By dynamically updating the smoothing intensity based on image error, we could avoid local minima and reduce divergence in both the camera and robot arm environments, as shown in Figure 5 and Figure 12. As the image error decreased, the gradient vanished. Therefore, weakening the smoothing intensity as the image error decreases effectively prevents the disappearance of the gradient. Moreover, updating the smoothing intensity allowed fine image features to be returned. This could initially increase the overall error, but subsequently decrease it. With dynamic intensity updates, the convergence was faster than with fixed smoothing. Certain divergence still occurred, probably before smoothing could be reduced. When the time constant parameter is adjusted to reduce the update speed, the smoothing duration increases. As Figure 12 shows, this resulted in a higher number of both divergent and successful cases. The time constant parameter is crucial for balancing local minima avoidance and convergence performance. Changing this significantly affects the performance, as shown in Figure 12. Further improvements in the performance may be achieved by fine-tuning this parameter in Equation (21).

Additionally, Equation (21) for the smoothing intensity proposed in this method takes a form similar to that of the free energy. However, this involves taking its reciprocal, which differs from the gradient update equation in the original free-energy principle. Therefore, there is room for discussion regarding its biological plausibility. Note that in human vision, the peripheral visual field is blurred, and the foveal vision is actively moved. The visual field updates from a blurred to a clear state [33]. The relationship between human eye movements and active inference has been highlighted in previous studies [34]. In this work, we restricted our comparison to conventional AIC and PB-AiF baselines that share the same generative model and inference scheme, in order to isolate the effect of the proposed adaptive smoothing. A systematic comparison with other techniques for escaping local minima, such as exploration-noise strategies (e.g., ϵ-greedy policies) or momentum-based optimizers, is left for future work. Furthermore, in the present study, the update rate *m* and the initial or final values of σ were manually tuned, and the performance can be sensitive to these choices. Developing principled, data-driven procedures for selecting or adapting these parameters across tasks is an important direction for future work.

In this study, we used uniform backgrounds for the experiments, but real-world scenarios often involve textured or cluttered ones. Gaussian smoothing blurs object boundaries, so when backgrounds aren’t uniform, it can mix the robot’s pixels with the background and severely harm state estimation. This is an important limitation for our approach. Moreover, the experiments in this study were conducted under idealized and simulated conditions that do not account for cases such as light reflections or the use of different robot arms, and the limitation that the performance depends on the image prediction error inherent in PB-AiF itself is also shared by our proposed method.

## 6. Conclusions

In this study, we propose a method for avoiding local minima in AIC by applying image smoothing to enhance their performance. The results showed that the proposed method outperforms conventional method in avoiding local minima. By dynamically adjusting the smoothing intensity based on free energy, the method effectively reduces divergence while maintaining robust local minima avoidance.

In the future, further performance improvements may be achieved by fine-tuning the time constant and updating the equation to smooth the intensity. The sensitivity of performance to smoothing intensity and time constant parameters warrants further investigation. While smoothing may not eliminate all local minima, some may prove beneficial. Extending the method to higher-dimensional and real-world environments is a future goal. Although this study focused on pixel-based active inference, the approach is also applicable to one-dimensional sensory data, potentially contributing to a broader framework for active inference.

## Figures and Tables

**Table 1:** Decoder model architecture.

No.	Layer Type	Kernel Size	Shape (Channel × Height × Width)
1	FC	-	512
2	FC	-	1024
3	FC	-	4096
4	Flatten	-	(16, 16, 16)
5	upConv	(4, 4)	(64, 32, 32)
6	Conv	(3, 3)	(128, 32, 32)
7	upConv	(4, 4)	(64, 64, 64)
8	Conv	(3, 3)	(64, 64, 64)
9	Dropout (*p* = 0.15)	-	(64, 64, 64)
10	upConv	(4, 4)	(1, 128, 128)

**Table 2:** Parameters used in the camera environment.

Posture	$\Sigma_{v}^{-1}$	$\Sigma_{\mu }^{-1}\beta$	$k_{v}$
*x*	80	6	0.2
*y*	80	4	0.2
θ	15	0.1	0.09

**Table 3:** Parameters used in the robot arm environment.

Joint	$\Sigma_{v}^{-1}$	$\Sigma_{\mu }^{-1}\beta$	$k_{v}$
1	150	0.001	$2\times 10^{-3}$
2	100	0.005	$5\times 10^{-3}$

## Data Availability

The datasets and related open-source resources used in this study are available through github (https://github.com/Kazuma0306/Python_Active_Inference, accessed on 16 December 2025).
